# Endocytosis of insulin at the blood-brain barrier

**DOI:** 10.3389/fddev.2022.1062366

**Published:** 2022-11-24

**Authors:** Sarah Pemberton, Demi C. Galindo, Michael W. Schwartz, William A. Banks, Elizabeth M. Rhea

**Affiliations:** 1 Geriatric Research Education and Clinical Center, VA Puget Sound Health Care System, Seattle, WA, United States; 2 Division of Metabolism, Endocrinology and Nutrition, Department of Medicine, School of Medicine, University of Washington, Seattle, WA, United States; 3 Division of Gerontology and Geriatric Medicine, Department of Medicine, School of Medicine, University of Washington, Seattle, WA, United States

**Keywords:** insulin transport, insulin receptor, endocytosis, microvessels, blood-brain barrier

## Abstract

For insulin to act within the brain, it is primarily transported from the blood across the blood-brain barrier (BBB). However, the endocytic machinery necessary for delivering insulin to the brain remains unknown. Additionally, there are processes within the brain endothelial cell that are designed to respond to insulin binding and elicit intracellular signaling. Using pharmacological inhibitors of different types of endocytosis (clathrin-vs. caveolin-mediated), we investigated molecular mediators of both insulin BBB binding in isolated mouse brain microvessels and BBB insulin transport in mice studied by brain perfusion. We found clathrin-mediated mechanisms responsible for insulin surface binding in isolated brain microvessels while caveolin-mediated endocytosis may mediate BBB insulin transport specifically in the hypothalamus. These results further define the molecular machinery necessary for transporting insulin into the CNS and highlight the distinction between insulin internalization for transendothelial transport vs. intracellular signaling.

## 1 Introduction

Insulin action in the central nervous system (CNS) is critically dependent on its transport across the blood-brain barrier (BBB). Once present in the CNS, insulin acts as a pleiotropic hormone, regulating metabolism, cognition, and mood. The BBB is a specialized structure comprised of endothelial and other cell types that tightly regulates substrate entry from blood to brain. At the BBB, paracellular transport is limited due to expression of tight junctions. Pinocytosis is also limited at this structure. While evidence for transendothelial insulin transport across the BBB has existed since the 1980s, the molecular processes involved within the brain endothelial cell (BEC) have not been largely investigated (Rhea and Banks, 2021; De Felice et al., 2022).

Transport of insulin across the BBB is unidirectional (from blood to brain) (Duffy and Pardridge, 1987; Schwartz et al., 1991; Cashion et al., 1996), occurs in a saturable manner (Schwartz et al., 1991; Banks et al., 1997a), can occur independent of the endothelial insulin receptor (Hersom et al., 2018; Rhea et al., 2018), and varies by brain region (Banks and Kastin, 1998). For example, the olfactory bulb displays the highest transport rate, nearly seven times greater than the whole brain transport rate (Banks et al., 1999). Additionally, there are regional differences in the expression of the insulin receptor throughout the brain (Rhea and Banks, 2021), which do not necessarily correlate with insulin brain transport. Therefore, it is highly possible that there are regional differences in transporter regulation and insulin receptor signaling. Due to the energy-dependent, saturable nature of insulin transport, insulin transport across the BEC has been assumed to be by transcytosis, requiring initiation of endocytosis on the luminal surface.

Endocytosis and transcytosis in BECs can occur *via* three currently known primary processes: clathrin-coated pits, caveolae, and macropinocytotic vesicles (Pulgar, 2018). In peripheral endothelial cells, the type of endocytosis process used for insulin transport varies across different vascular beds. For example, clathrin-mediated endocytosis is the main form of endocytosis in the microvasculature (Azizi et al., 2015) whereas in the macrovasculature, such as the aorta, caveolin-1 is the primary mediator (Wang et al., 2011). This observation suggests that mechanisms underlying transendothelial insulin transport vary across tissues and vascular beds (Lee and Klip, 2016). The BBB is present in arterioles, capillaries, and venules throughout the brain, differentially expressing a wide range of genes based on anatomical axis, recently described as cellular zonation (Vanlandewijck et al., 2018). The majority of nutrient exchange occurs at the capillary level.

We previously reported that in mice, transendothelial insulin transport is minimally impacted by either targeted deletion or pharmacological blockade of the endothelial insulin receptor. In an effort to identify an insulin receptor-independent transport mechanism for insulin, we investigated whether either clathrin- or caveolin-mediated mechanisms are involved (Rhea et al., 2018). Most investigations for endocytosis in BECs regarding insulin have been *in vitro* including cell culture systems and isolated brain microvessels (Frank and Pardridge, 1981; Frank et al., 1986; Gray et al., 2017). While these models are useful for molecular investigations, they also have limitations that need to be taken into consideration, such as high levels of insulin present in the culture media and lack of signals coming from other cells of the neurovascular unit including astrocytes and pericytes (Rhea and Banks, 2021). The molecular mechanisms involved in BEC endocytosis of insulin *in vivo* remain to be investigated. Understanding this pathway could aid in improving insulin BBB transport in CNS insulin dysregulated conditions such as obesity and Alzheimer’s disease (Rhea and Banks, 2019). Here, we investigate molecular mediators of insulin BBB transport and binding in isolated mouse brain microvessels as well as in mice following cardiac perfusion.

## 2 Materials and methods

### 2.1 Animals

Six-week old male CD-1 mice were purchased from Charles River Laboratories (Seattle, WA, United States) and kept on a 12-h light/dark cycle with *ad libitum* access to food and water. Mice were studied by 8 weeks of age. Prior to microvessel isolation, mice were cervically dislocated. Prior to *in vivo* studies, mice were anesthetized with an intraperitoneal (ip) urethane (40%) injection (0.15 ml). All protocols were approved by the VAPSHCS Institutional Animal Care and Use Committee and performed at a facility approved by the Association for Assessment and Accreditation of Laboratory Animal Care International.

### 2.2 Inhibitors

All endocytosis inhibitors were supplied from Sigma-Aldrich. Monensin and chlorpromazine (CPZ) inhibit clathrin-mediated endocytosis. Monensin inhibits clathrin-dependent endocytosis by dissipating a proton gradient (Dickson et al., 1982). CPZ causes clathrin to translocate to intracellular endosomes, depleting it from the plasma membrane (Wang et al., 1993). Filipin III was used to inhibit caveolae-mediated endocytosis. Filipin acts by disassembling endothelial non-coated plasmalemmal vesicles, also known as lipid rafts (Schnitzer et al., 1994). S961 is a selective antagonist for the insulin receptor (Schaffer et al., 2008) and was graciously provided by Professor Lauge Schaffer at Novo Nordisk (Denmark).

### 2.3 Radioactive labeling of peptides

Ten μg human insulin (Sigma-Aldrich, St Louis, MO, United States), the insulin receptor antagonist S961 (Novo Nordisk, Denmark), or human holo-transferrin (Sigma-Aldrich) was diluted in 0.25 M sodium phosphate buffer (PB, pH 7.5), and the chloramine-T (Sigma-Aldrich) method was used to radioactively label the peptides with 1 mCi Na^125^I (Perkin-Elmer, Waltham, MA, United States) (Rhea et al., 2018). The reaction started with the addition of 10 μg chloramine-T in 0.25 M PB and terminated 1 min later with addition of 100 μg sodium metabisulfite. ^131^I-albumin was labeled was labeled the same way except used 2 mCi ^131^I (Perkin-Elmer, Waltham, MA, United States). ^131^I-albumin was used as a vascular marker for the ^125^I-transferrin multiple-time regression analysis transport studies as previously described (Rhea et al., 2018). A Sephadex G-10 column (Sigma-Aldrich) was used to separate labeled peptides and proteins from free iodine. Protein labeling was characterized by 15% trichloroacetic acid (TCA) precipitation. The radioactively labeled substrates we injected were quality-controlled materials, negating the overestimation of free ^125^I.

### 2.4 Microvessel isolation and processing

Whole brains were pooled and homogenized in chilled isolation buffer (DMEM, 25 mM HEPES, pH 7.4, 1% (w/v) dextran and 0.5% BSA) with a Dounce homogenizer. A range of 8–20 brains were pooled each experimental day. The homogenate was poured once through a 300 μm mesh, then twice through a 100 μm mesh. The filtrate was combined with an equivalent volume of chilled 40% dextran in isolation buffer and centrifuged at 3000 g for 30 min at 4°C. The pellet was resuspended in 1 ml of isolation buffer and pipetted twice through a 20 μm pluriStrainer^®^ mesh (pluriSelect Life Science, Leipzig, Germany), before a final rinse with isolation buffer. The microvessels residing on the surface of the 20 μm mesh were collected in isolation buffer, and the sample was centrifuged at 2000 g for 15 min at 4°C. The pellet was resuspended in 1 ml of PBS and spun at 500 g for 3 min at 4°C three times. The final pellet was resuspended in incubation buffer (129 mM NaCl, 2.5 mM KCl, 7.4 mM Na_2_HPO_4_, 1.3 mM KH_2_PO_4_, 0.63 mM CaCl_2_, 0.74 mM MgSO_4_, 5.3 mM D-glucose, 0.1 mM ascorbic acid, 1% BSA, pH 7.4) before the microvessel binding assay.

Isolated microvessels were processed as previously described (Banks et al., 1997b; Banks et al., 2001) with a few modifications. Briefly, the suspended pellet was evenly split between treatment groups, with two technical replicates per group. On average, each binding assay involved approximately 90 μg protein from the isolated microvessels in 40–45 μL. Each study was repeated with separate isolated microvessels for 2-3 biological replicates per study. Microvessels were treated with vehicle or an endocytosis inhibitor. Inhibitors were made up in incubation buffer and/or MeOH. Final concentrations for inhibitors include: monensin (10 μM), CPZ (2.8 mM in 20% MeOH), and filipin III (2 μg/ml in 0.2% MeOH) with respective vehicle MeOH controls. These doses were chosen based on previous *in vitro* and *in vivo* studies (Schnitzer et al., 1994; Banks et al., 1997b; Turinsky et al., 1997). Samples were incubated for 15 min at 37°C (Banks et al., 2001), then treated with ^125^I-insulin (400,000 CPM in incubation buffer) and incubated for an additional 15 min at 37°C. Samples were centrifuged at 10,000 g for 2 min at 4°C, and the supernatants were collected. The pellets were resuspended in incubation buffer, centrifuged at 4,225 g for 2 min at 4°C, and the supernatants were combined with the previous ones. The radioactivity in the pellets and combined supernatants were measured in a gamma counter (Wizard^2^, PerkinElmer). In order to determine the amount of substrate bound to the cell surface, the pellet was resuspended in 400 μL of chilled acid wash buffer (0.2 M glycine, 0.15 M NaCl) to strip the surface of reversibly bound substrates on ice for 6 min, centrifuged at 4,225 g for 2 min at 4°C. The supernatant was collected and measured in a gamma counter as before to determine the amount of reversible binding (RB) at the plasma membrane, which we have called surface binding. To determine the amount of substrate internalized, the pellet was resuspended in 400 μL of chilled 1% BSA in deionized water and incubated for 1 h on ice to lyse the cells. The sample was centrifuged as before, the supernatant was collected, and the pellet was suspended again in 1% BSA and incubated on ice for 30 min. The sample was centrifuged, the supernatant was combined with the previous one, and both supernatant and pellet were measured in a gamma counter. As we were interested in the total amount of insulin internalized, regardless of whether it was still bound to internal membranes or in the cytosol, we measured both the cytoplasmic fraction (C, the final supernatant), and the internalized membrane fraction (M, the pellet). Calculations are as follows:

Total Counts Internalized(I)=C+M

Percentages were calculated by dividing the measurement of interest (surface binding or internalization) by the total counts present in the sample and multiplying by 100. Treated samples were made relative to vehicle controls within each day-of experiment.

### 2.5 *In vivo* S961 binding

In anesthetized mice, the jugular vein was exposed and a 100 μL IV injection containing vehicle (10% MeOH in 0.1% BSA/LR) or monensin (50 μM in 10% MeOH in 0.1% BSA/LR) was administered. Thirty min later, a second 100 μL IV injection containing 1 × 10^6 ^125^I-S961 was administered and circulated for 5 min. Blood was collected from the descending aorta, the olfactory bulbs and whole brains removed, and whole brains dissected into 10 brain regions according to the method by Glowinski and Iversen, (1966). The amount of radioactivity in each region was counted in a gamma counter (Wizard^2^, PerkinElmer) and corrected for the % injected ^125^I-S961 (based on injection checks) and divided by the weight of the tissue (g) to get %Inj/g levels.

### 2.6 *In situ* brain perfusion

Following anesthesia, the thoracic cavity was opened and the descending thoracic aorta was clamped and jugular veins were cut to allow blood to drain from the brain vascular space. A 26-gauge butterfly needle was inserted into the left ventricle of the heart and mice were pre-perfused with vehicle or inhibitor at a rate of 2 ml/min. Mice were perfused with vehicle (1% MeOH in Zlokovic buffer: 7.19 g/L NaCl, 0.3 g/L KCl, 0.28 g/L CaCl_2_, 2.1 g/L NaHCO_3_, 0.16 g/L KH_2_PO_4_, 0.17 g/L anhydrous MgCl_2_, 0.99 g/L D-glucose and 1% BSA) ± 5 μM monensin or 2 μg/ml filipin III. After a 10 min pre-treatment with the inhibitor, ^125^I-insulin (2 × 10^5^ cpm/mL) was perfused through the left ventricle of the heart at a rate of 2 ml/min. Mice were perfused for timepoints ranging from 0.5 to 5 min. Whole brains, olfactory bulbs, and the hypothalamus were collected, weighed, and measured for radioactivity in a gamma counter. Brain/perfusate ratios (μL/g) were calculated by dividing the cpm per Gram of tissue by the cpm per μL of perfusate and plotted against perfusion time.

### 2.7 Statistics

Regression analysis and other statistical analyses were performed using Prism 8.0 (GraphPad Software Inc., San Diego, CA, United States). For microvessel binding assays, the means are reported with their standard error terms and compared by a Student’s t test was. *p* values less than 0.05 were considered statistically significant. For *in situ* brain perfusion studies, linear regressions were performed on the brain/serum or brain/perfusate ratios using GraphPad Prism software. Linear regression lines were compared statistically with the Prism 8.0 software package. They are reported with their correlation coefficients (*r*) and linear regression *p* values.

## 3 Results

### 3.1 *In vitro* insulin binding and endocytosis

**FIGURE 1:**
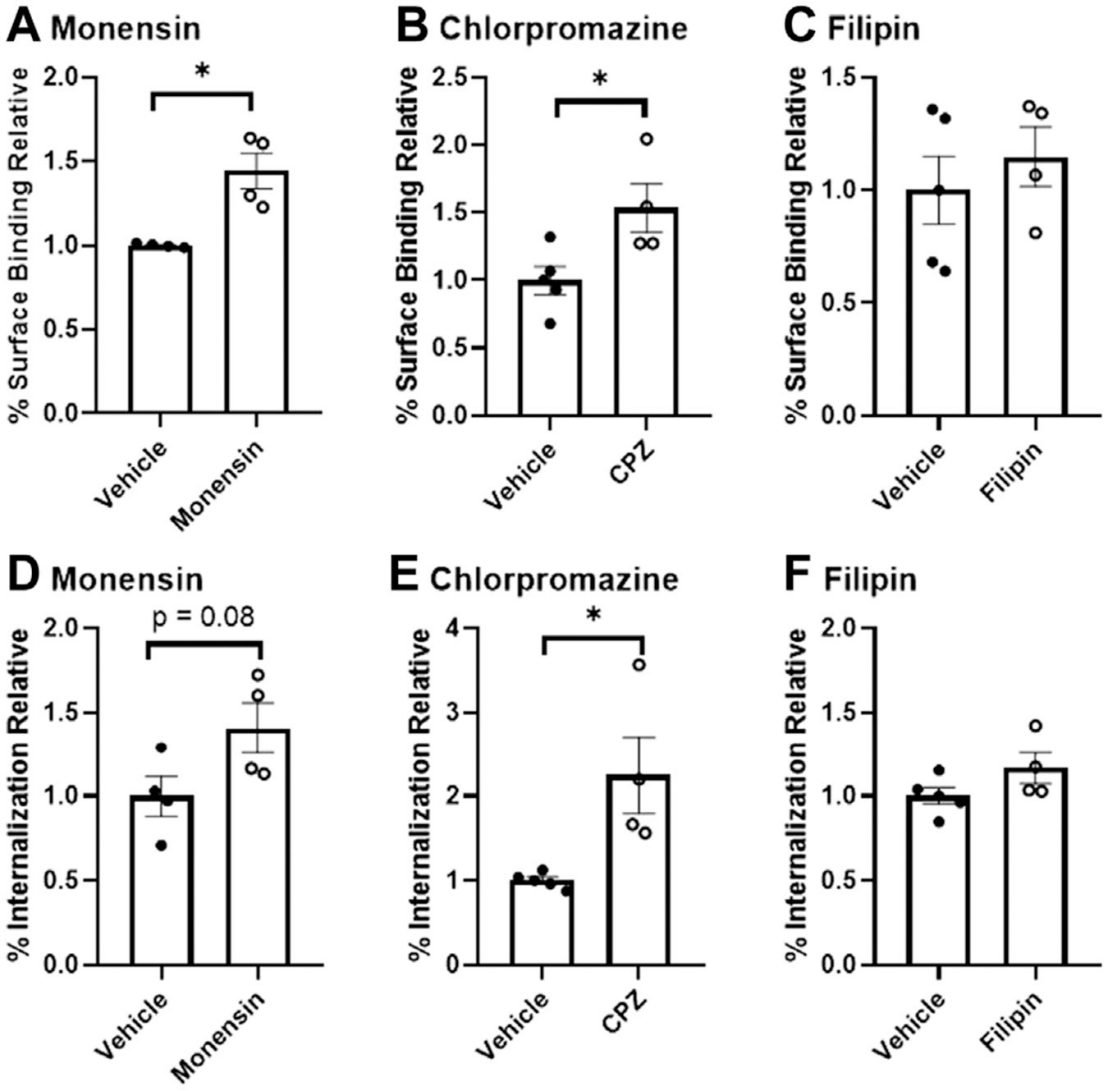
Binding and endocytosis of insulin in isolated mouse brain microvessels. Surface Binding **(A–C)** and Internalization **(D–F)** of ^125^I-insulin was investigated. ^125^I-insulin surface binding was significantly increased in the presence of monensin and chlorpromazine (*p < 0.05), but not filipin. The amount of ^125^I-insulin internalized was significantly increased with E) chlorpromazine (*p < 0.05).

Using assays of radiolabeled insulin binding and uptake in pooled, isolated murine brain microvessels, we report that inhibitors of clathrin-mediated endocytosis (monensin and chlorpromazine (CPZ)) caused a significant increase in the microvessel surface binding of ^125^I-insulin (Figure 1A, *p* = 0.006, 36.3% difference for monensin and Figure 1B, *p* = 0.031, 42.2% difference with CPZ). CPZ treatment induced a 76.9% increase in the amount of insulin internalized (Figure 1E, *p* = 0.018). Monensin did not produce a statistically significant change in internalization, but the results were trending (Figure 1D, *p* = 0.079, 33.7% difference). Inhibition of caveolin did not have any effect on endocytosis (Figures 1C,F). Altogether, these findings suggest that insulin binding and endocytosis in isolated brain microvessels is regulated by clathrin and not by caveolin.

### 3.2 *In vivo* S961 binding following clathrin inhibition

**FIGURE 2:**
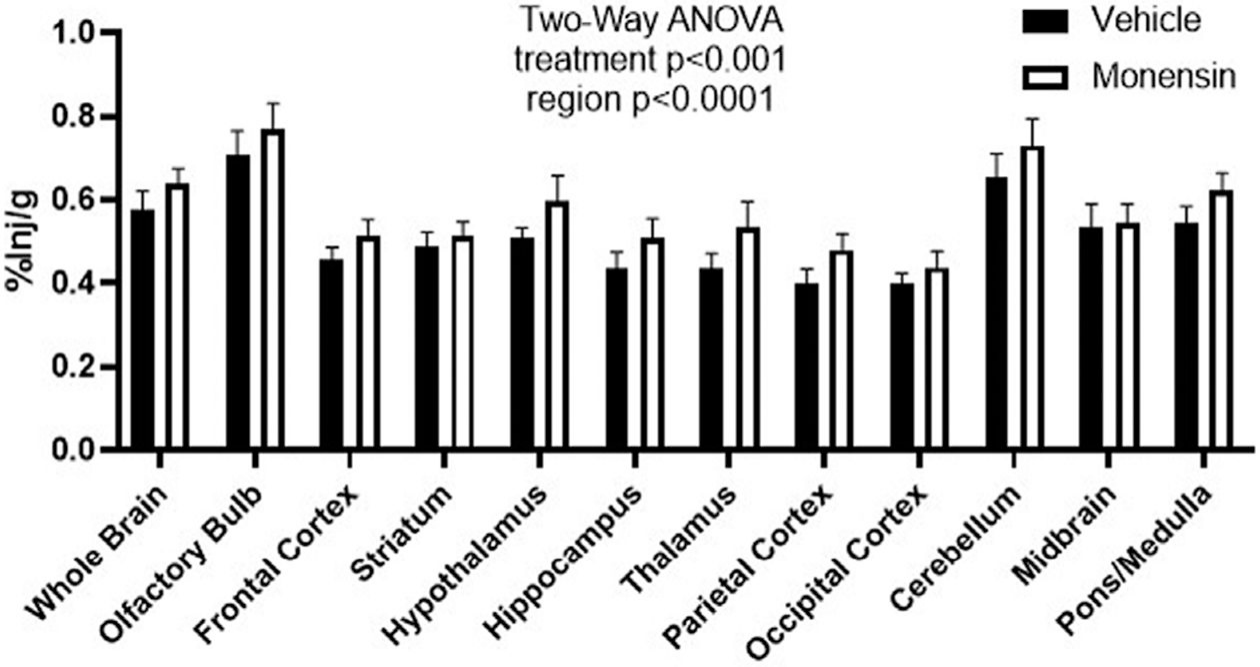
Regional binding of ^125^I-S961 *in vivo* following clathrin inhibition. ^125^I-S961 binding at the BBB was increased following a 30 min pre-treatment IV injection of 50 μM monensin compared to vehicle (10% MeOH in 0.1% BSA/LR). Two-way ANOVA treatment *p* < 0.001, no post hoc differences. *n* = 6/group.

To investigate whether monensin pre-treatment *in vivo* could impact insulin receptor surface binding, we utilized ^125^I-S961, which binds to the insulin receptor with high affinity. We have previously shown ^125^I-S961 does not cross the BBB (Rhea et al., 2018), but rather acts as a marker for surface binding. Here, we report that following a 30 min pre-treatment with IV monensin to block clathrin-mediated endocytosis, ^125^I-S961 binding (%Inj/g) was significantly increased (two-way ANOVA treatment *p* = 0.0008, Figure 2). There were no post-hoc differences. These data support our *in vitro* studies showing a role for clathrin-mediated endocytosis in insulin receptor recycling in BECs.

### 3.3 *In situ* insulin transport following endocytosis inhibition

**FIGURE 3:**
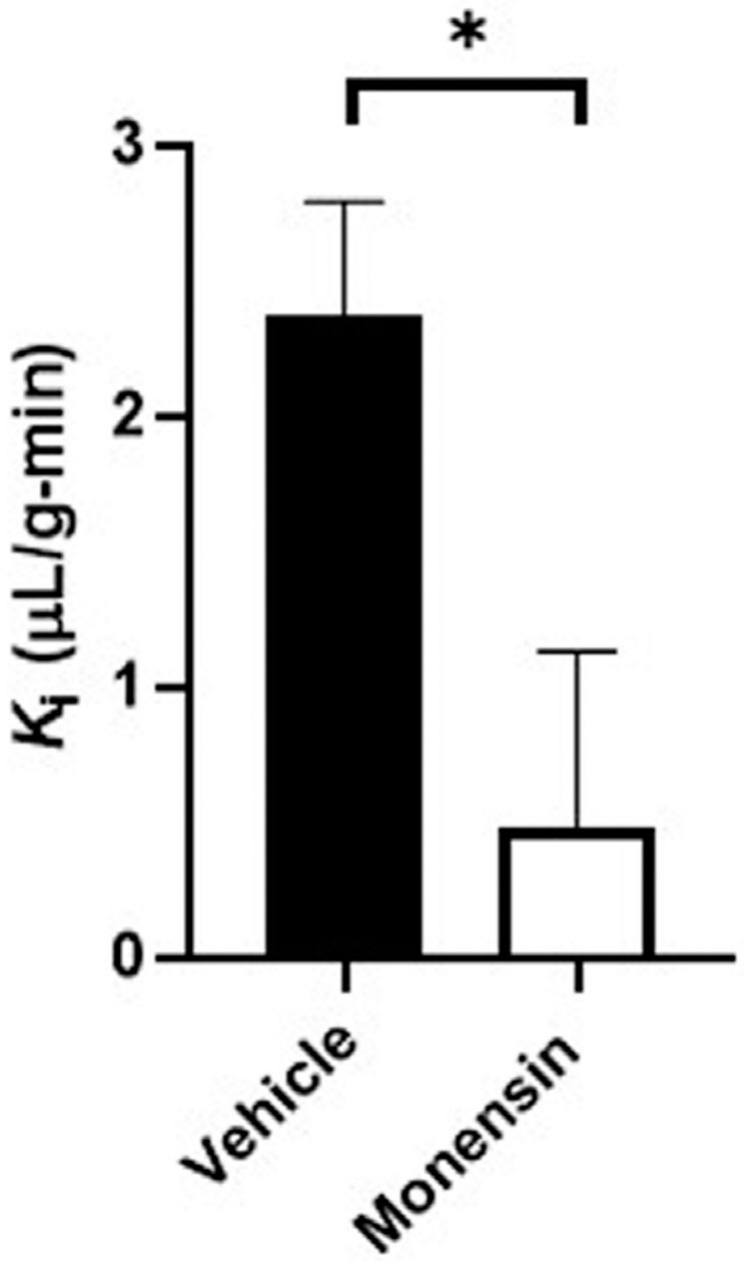
Role of clathrin in transferrin hypothalamic BBB transport. An IV pre-treatment (30 min) with 50 μM monensin (in 10% MeOH in 1% BSA/LR) was able to significantly decrease hypothalamic ^125^I-transferrin transport (K_*i*_), following correction of the vascular marker, ^131^I-albumin, *in vivo*. *n* = 8/group.

To validate the use of the clathrin inhibitor *in vivo*, we first investigated transferrin BBB transport, a substrate shown by ultrastructural electron microscopy to involve clathrin (Roberts et al., 1993). Mice pre-treated with monensin resulted in a significant 80% decrease in the hypothalamic BBB transport of ^125^I-transferrin (Veh K_*i*_ = 2.37 ± 0.43 vs. Monensin K_*i*_ = 0.48 ± 0.66 μL/g-min, *p* = 0.033, Figure 3) *in vivo* following correction of the vascular space. These data provide additional evidence of the effect of monensin to block clathrin-mediated BBB transendothelial transport *in vivo* and acts as a positive control for our insulin transport investigations.

**FIGURE 4:**
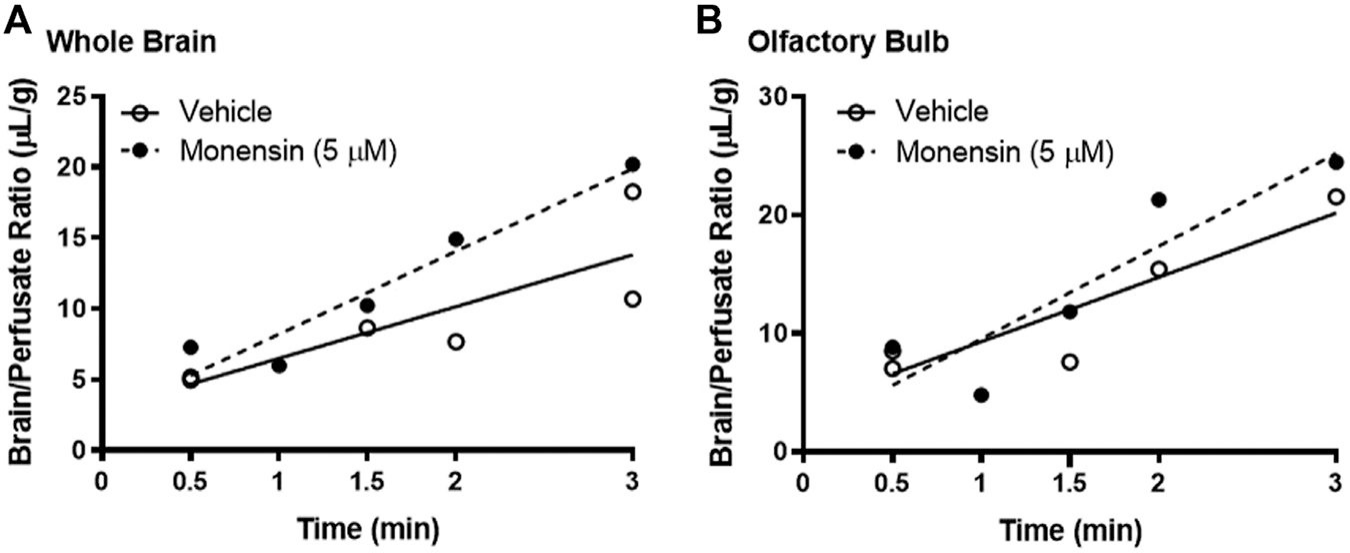
Role of clathrin in insulin BBB transport. Pre-treatment (10 min) with 5 μM monensin has no effect on ^125^I-insulin transport (K_*i*_) or vascular binding (V_*i*_) in **(A)** whole brain or **(B)** olfactory bulb. Table 1 lists the K_*i*_ and V_*i*_ for each linear regression.

To eliminate the effects of circulating serum factors and more directly investigate endocytosis machinery at the BEC surface, we pre-treated mice with *in situ* brain perfusions of endocytosis inhibitors. First, to investigate the role of clathrin, we pre-perfused 5 μM monensin *via* cardiac perfusion. After 10 min, we perfused ^125^I-insulin to measure BBB transport. Neither whole brain nor olfactory bulb displayed a significant change in insulin transport (K_i_) or endothelial cell binding (V_i_) when clathrin was inhibited (Figure 4; Table 1). We found similar results when mice were pre-treated intravenously with monensin 30 min before insulin BBB transport investigation by cardiac perfusion (data not shown). Unlike transferrin, therefore, insulin transport across the BBB does not appear to be clathrin-dependent.

**FIGURE 5:**
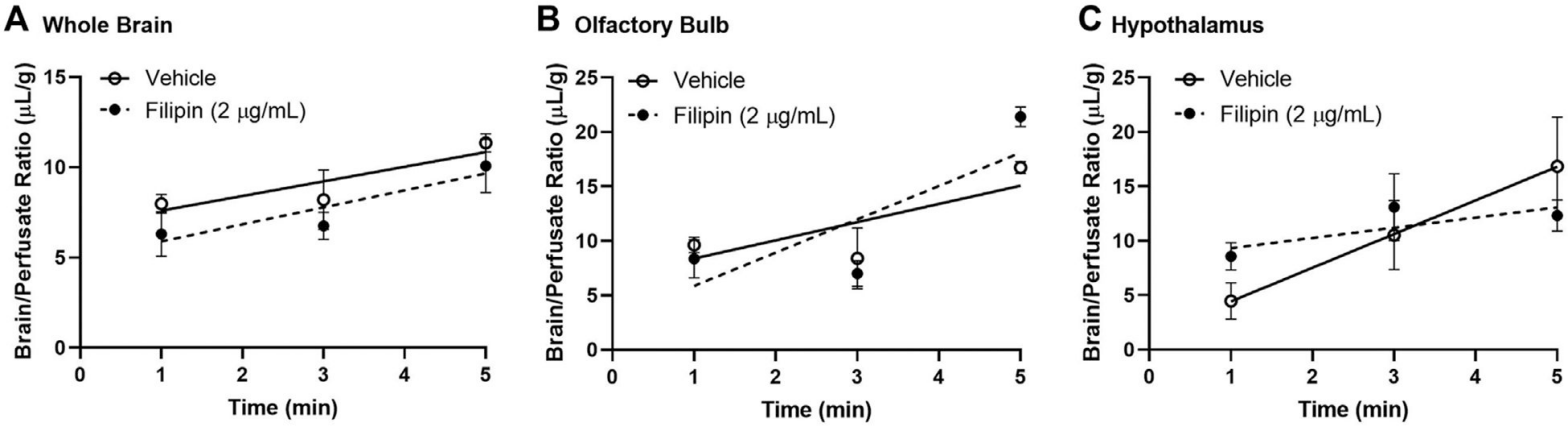
Role of caveolin in insulin BBB transport. Pre-perfusion for 10 min with 2 μg/ml filipin has no effect on ^125^I-insulin transport (K_*i*_) or vascular binding (V_*i*_) in **(A)** whole brain or **(B)** olfactory bulb but does limit transport (K_*i*_) in the **(C)** hypothalamus. Table 2 lists the K_*i*_ and V_*i*_ for each linear regression.

We then measured the impact of blocking caveolin-mediated endocytosis using the same methods, substituting filipin (2 μg/ml) for monensin pre-treatment prior to ^125^I-insulin perfusion (Figure 5). Caveolin inhibition did not impact insulin uptake or binding in either the whole brain or the olfactory bulb (Figures 5A,B; Table 2). In the hypothalamus, however, filipin pre-treatment reduced the rate of insulin transport markedly, to a value that did not differ from zero (ns) (Figure 5C; Table 2, K_i_ (ns) = 0.94 ± 0.43 μL/g-min, *p* = 0.179) compared to the vehicle control (K_i_ = 3.1 ± 0.99 μL/g-min, *p* = 0.014). This evidence that hypothalamic BBB insulin transport is blocked by filipin suggests that insulin transcytosis in this specific brain area is dependent on caveolin. Thus, mechanisms underlying insulin transport across the BBB may vary depending on brain region.

## 4 Discussion

**FIGURE 6:**
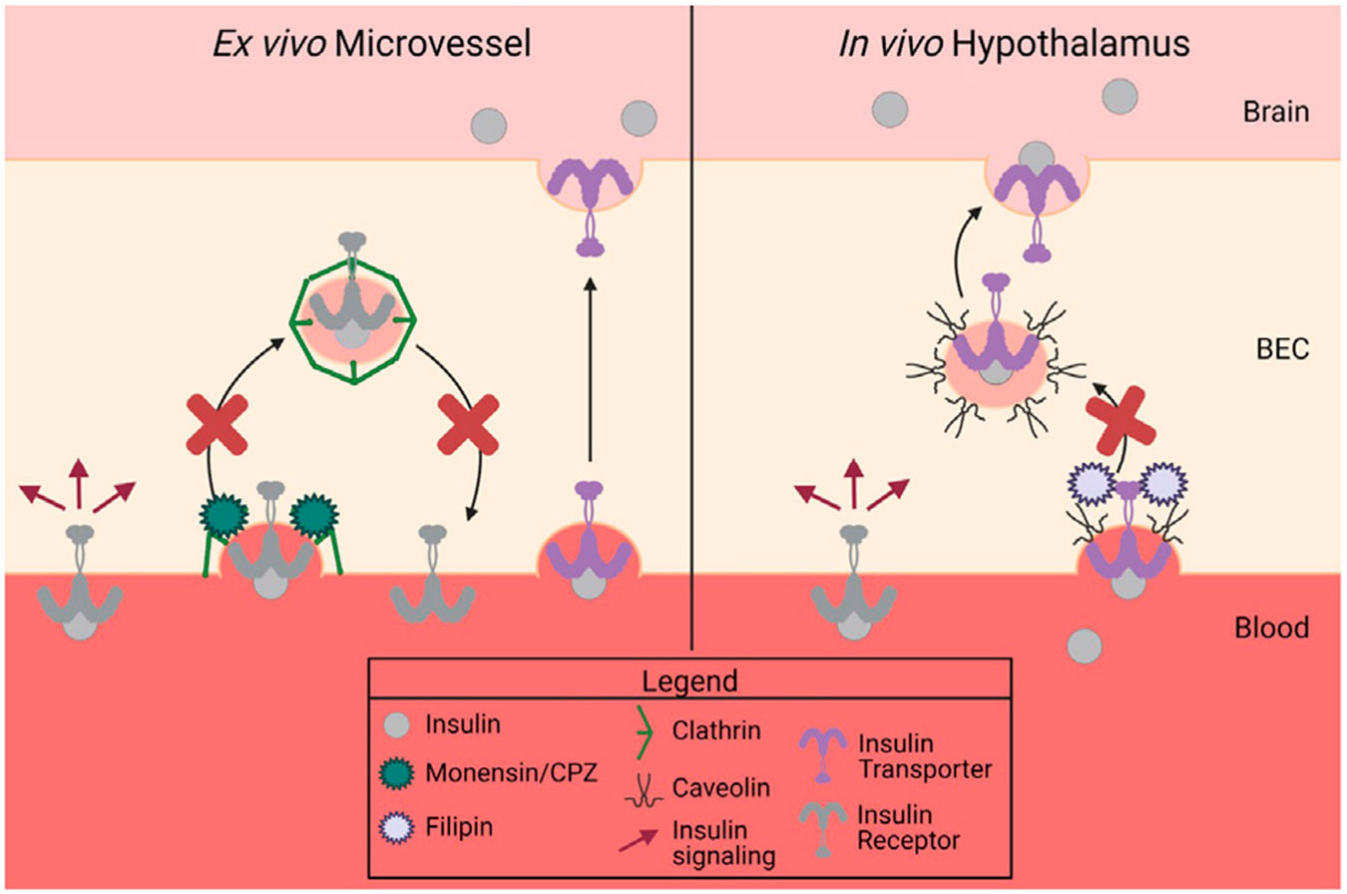
Summary of data. There are two separate proteins responsible for insulin binding (receptor) and insulin transport (transporter) at the BBB. Insulin binding on isolated mouse microvessels is clathrin-dependent (left side), while *in vivo* insulin transport is regionally regulated but dependent on caveolin in the hypothalamus (right side). This figure was created with Biorender.com.

Due to our previous report identifying an insulin receptor-independent transport mechanism for insulin at the BBB (Rhea et al., 2018), we investigated whether clathrin- or caveolin-mediated mechanisms are involved in insulin binding or transport. Using previous well-described methods for investigating substrate binding in isolated mouse brain microvessels and substrate transport across the BBB *in vivo via* the cardiac perfusion method, our findings extend previous literature which have investigated the endocytosis machinery necessary for insulin transport in other vascular beds (King and Johnson, 1985; Azizi et al., 2015). We report that clathrin-mediated endocytosis is primarily involved in insulin surface binding in isolated brain microvessels while caveolin-mediated endocytosis may be involved in insulin transport specifically in the hypothalamus (Figure 6).

Our previous findings that BBB insulin transport involves mechanisms that are at least in part independent of the insulin receptor (Rhea et al., 2018) suggests the existence of a distinct but as yet unidentified insulin transport mechanism. Previous studies utilizing isolated brain microvessels have shown that insulin binding is competitive and internalization is energy dependent (Frank et al., 1986). Based on these findings, we measured insulin binding and internalization in BECs from isolated microvessels *ex vivo*. We found that clathrin inhibition *via* either monensin or CPZ increased insulin binding on surface of BECs. This suggests clathrin is required for insulin binding to BECs. Whether the increased binding on the BEC surface represent insulin receptor binding sites or binding to the insulin transporter cannot be differentiated in this study. Secondly, we inhibited caveolin endocytosis in isolated microvessels and found that there was no effect on insulin binding or internalization. Therefore, we can conclude that cell surface binding of insulin in isolated brain microvessels is regulated by clathrin, as it is in other microvascular endothelial cells (Azizi et al., 2015), and not caveolin. Previous studies in endothelial cells have shown that the peripheral insulin receptor is mainly or partially localized in plasma membrane caveolae (Stralfors, 2012). However, our data suggests in isolated brain microvessels, insulin receptor surface localization is likely regulated by clathrin. Again, this data supports endothelial vascular beds can be quite different regarding insulin receptor internalization. In contrast, others have shown that caveolin-1 inhibition decreases insulin uptake in a commercially available *in vitro* BEC model (Gray et al., 2017). However, these *in vitro* models are difficult to investigate BBB functionality due to the high levels of insulin present in the culture media and lack of contribution from other cell types of the neurovascular unit astrocytes, pericytes.

We also found that clathrin inhibition increased the amount of insulin internalized into brain microvessels. This initially seemed puzzling as we were not able to explain both an increase in insulin binding, concomitant with an increase in insulin internalization by the same mechanism. However, it is possible that the increase in insulin internalization was due to compensation by caveolin-mediated insulin internalization. That is, when clathrin-mediated endocytosis was unavailable, caveolar endocytosis took over. Since we cannot measure transcytosis in isolated brain microvessels, we moved to our well-established *in vivo* and *in situ* techniques to measure insulin BBB transport.

Next, we wanted to determine *in vivo*, what type of endocytosis machinery was necessary for insulin transcytosis at the BBB. In order to allow the inhibitor to work directly at the BBB, we pre-perfused the inhibitors to allow the cells to respond prior to the insulin transport assay. Following exposure to the endocytosis inhibitor, we perfused radioactive insulin for different lengths of time to calculate the transport rate across the BBB. While we did not see an effect with monensin on insulin pharmacokinetics, we found that filipin limited insulin uptake at the hypothalamic BBB. This suggests that caveolin-mediated endocytosis plays a role in insulin transport across the BBB. Additionally, we only saw this effect in the hypothalamus and not in the olfactory bulb or whole brain. This suggests that the transport of insulin across the BBB is regulated differently amongst the brain regions (Rhea et al., 2019) and warrants further investigation. Since insulin BBB transport was not completely abolished by inhibition of caveolin-1, it suggests there are potentially other transport mechanisms in play, providing a back-up system in case one becomes dysfunctional. Indeed, the hypothalamus contains unique transport processes, including the presence of the tanycyte barrier, which makes this region unique amongst other brain regions (Rodriguez et al., 2010). We recognize that these inhibitors can affect molecules other than their primary target (Dutta and Donaldson, 2012). Filipin, in particular, binds to cholesterol and so may impact other forms of lipid domain endocytosis besides the caveolin-mediated pathway. In order to validate our findings, it would be important to repeat this study in mice that have clathrin- or caveolin-targeted knockdown through gene silencing techniques.

The idea that insulin transport at the BBB could be regulated differently in distinct brain regions is reasonable. A recent paper investigating the impact of peripheral insulin infusion on CNS gene regulation showed that brain regions responded quite differently to the insulin (Cai et al., 2021). For example, the hypothalamus, an area, that is, important in regulating metabolism and has one of the higher levels of insulin transport (Rhea et al., 2018), has a robust change in gene expression when exposed to peripheral insulin. The hippocampus, important for memory formation, on the other hand, has about a 1/10th of the response to peripheral insulin on gene regulation. Transport of insulin into this region, by comparison, is approximately half that of the hypothalamus. These studies demonstrate transport can be altered depending on the physiological need at any given time.

In conclusion, we have demonstrated insulin endocytosis in isolated mouse brain microvessels occurs in a clathrin-dependent manner. On the other hand, we showed *in situ*, insulin transcytosis at the BBB varies regionally and can be mediated by caveolin at the hypothalamic BBB. Our study is limited as we restricted our binding and transport studies to only investigating the two main forms of endocytosis. However, there are other forms of endocytosis that could be involved in insulin BBB transcytosis (Doherty and McMahon, 2009), or insulin BBB transport could rely on a process other than endocytosis. Models using a more specific knock down of these processes or investigating other forms of transcytosis is warranted to further investigate insulin BBB transport molecular machinery.

## Figures and Tables

**TABLE 1:** BBB pharmacokinetics of ^125^I-insulin during clathrin inhibition.

Region	Treatment	K_*i*_ (μL/g min)	*p*	r	V_*i*_ (μL/g)
Whole Brain	Vehicle	3.66 ± 1.2	0.037	0.84	2.85 ± 2.4
	Monensin	5.84 ± 1.0	0.009	0.96	2.37 ± 1.8
Olfactory Bulb	Vehicle	5.44 ± 1.4	0.029	0.92	3.87 ± 2.5
	Monensin	7.83 ± 2.2	0.036	0.90	1.71 ± 3.9

**TABLE 2:** BBB pharmacokinetics of ^125^I-insulin during caveolin inhibition.

Region	Treatment	K_*i*_(μL/ g min)	p	r	V_*i*_ (μL/g)
Whole Brain	Vehicle	0.81 ± 0.34	0.045	0.64	6.8 ± 1.1
	Filipin	0.94 ± 0.43	0.048	0.54	5.0 ± 1.5
Olfactory Bulb	Vehicle	1.67 ± 0.68	0.039	0.66	6.7 ± 2.2
	Filipin	3.06 ± 0.93	0.010	0.74	2.8 ± 3.0
Hypothalamus	Vehicle	3.10 ± 0.99	0.014	0.74	1.3 ± 3.2
	Filipin	0.94 ± 0.66 (ns)	0.179	0.38	8.4 ± 2.3

ns, slope did not significantly differ from zero.

## Data Availability

The original contributions presented in the study are included in the article/supplementary material, further inquiries can be directed to the corresponding author.
